# Phenotypic Profiling of Immune Cells and Their Mediators in Chronic Obstructive Pulmonary Disease

**DOI:** 10.3390/biomedicines11082166

**Published:** 2023-08-01

**Authors:** Meghashree Sampath, Geetanjali Bade, Randeep Guleria, Anant Mohan, Sudip Sen, Devanjan Dey, Anjana Talwar

**Affiliations:** 1 Department of Physiology, All India Institute of Medical Sciences, New Delhi 110029, India; meghashree90@gmail.com (M.S.); geetanjalibade@gmail.com (G.B.); 2 Department of Pulmonary, Critical Care and Sleep Medicine, All India Institute of Medical Sciences, New Delhi 110029, India; randeepg@hotmail.com (R.G.); anantmohan@yahoo.com (A.M.); 3 Department of Biochemistry, All India Institute of Medical Sciences, New Delhi 110029, India; sudipsen665@gmail.com (S.S.); devanjandey@gmail.com (D.D.)

**Keywords:** COPD, immunophenotyping, T cells

## Abstract

Background: Chronic obstructive pulmonary disease (COPD) is an inflammatory disorder and has been proposed to have an imbalance between pro-inflammatory and anti-inflammatory factors. Methods: This study was conducted on 41 participants {18 COPD patients (smokers, COPD S (*n* = 9); reformed smokers, COPD RS (*n* = 9)) and 23 controls (non-smokers, CNS (*n* = 14); smokers, CS (*n* = 9))}. Flow cytometry was used to identify circulatory immune cells and correlated with serum cytokines. Results: On comparison, significantly lower frequency of CD3+ T cells were observed in COPD S as compared to CNS (*p* < 0.01) and CS (*p* < 0.01); CD4+ T cells were lower in COPD S (*p* < 0.05), COPD RS (*p* < 0.05) and CNS (*p* < 0.01) as compared to CS. CD8+ T cells were elevated in COPD S as compared to CS (*p* < 0.05). Lower frequency of cDCs were observed in COPD S as compared to CS (*p* < 0.05) and COPD RS as compared to CNS (*p* < 0.01) and CS (*p* < 0.01). Lower frequency of pDCs were observed in COPD RS as compared to COPD S (*p* < 0.05), CNS (*p* < 0.05) and CS (*p* < 0.01). Lower frequency of Tregs was observed in COPD S as compared to CNS (*p* < 0.05) and CS (*p* < 0.05). Conclusions: Characteristic changes observed indicate a significant impact of immune cells in the progression of the disease.

## 1. Introduction

Chronic obstructive pulmonary disease (COPD) is a prevalent, treatable, chronic lung disease characterized by persistent respiratory symptoms and airflow limitation caused primarily by prolonged exposure to noxious particles or gases [1]. The global prevalence of COPD was 10.3% in 2019, accounting for 391.9 million cases among adults aged 30–79 years using the Global Initiative for Obstructive Lung Disease (GOLD) definition [1,2]. COPD is the consequence of a complex interaction of chronic cumulative exposure to smoke or noxious particles, combined with an array of host factors like genetic predisposition, airway hyperresponsiveness or environmental pollution including biomass combustion. Though COPD is primarily caused by cigarette smoke, despite smoking cessation, the restorative processes of the airway structure and function do not completely revert to its normal state. In fact, there is an amplification of the disease process in COPD patients who are either smokers and/or ex-smokers as compared to smokers who do not have airflow limitation.

There is a distinct pattern of inflammation in these patients, with increased numbers of neutrophils in the airway lumen as well as macrophages, T cells and B lymphocytes; it involves innate immune cells, adaptive immune cells, as well as the activation of structural cells, endothelial cells and fibroblasts.

Patients with COPD have been observed to have systemic inflammation, characterized by increased levels of circulating cytokines, chemokines and acute-phase proteins and/or abnormalities in peripheral immune cells, especially in severe disease and during acute exacerbations [3]. The inflammatory pattern that accelerates the exacerbation process might be a consequence of AIM2-dependent oxidative stress which has been reported to be unaffected by corticosteroid therapy [4,5]. COPD being an inflammatory disorder has been proposed to have an imbalance between pro-inflammatory and anti-inflammatory factors. Previous studies have revealed aberrant immune cells and their mediators in COPD patients as compared to controls [6,7].

To the best of our knowledge, the literature regarding relative numbers of Tregs and their interaction with other immune cells in peripheral circulation of COPD patients remains unsettled.

This proposed study thus aims to investigate the relative numbers of circulatory immune cells and their associated cytokines in patients with COPD. Investigating the baseline profile of immune cells in the disturbed immune homeostasis of COPD may provide an opportunity to gain better and deeper insights into the pathophysiological basis of the disease.

## 2. Materials and Methods

### 2.1. Participants

All the participants were recruited after prior diagnosis by practicing clinicians in the Department of Pulmonary, Critical Care and Sleep Medicine. Written informed consent as per Helsinki Declaration (amendment 2013) was obtained from all participants prior to their inclusion in this study. This study was approved by the Institute ethical committee (IECPG-115/26.04.2017, RT-09/2017 dated 14 August 2017) for human subjects. Participants were divided into two groups, i.e., patients and controls, which were further subcategorized into COPD smokers (COPD S), COPD reformed smokers (COPD RS), control non-smokers (CNS) and control smokers (CS), respectively.

Clinically diagnosed male COPD patients having mild to moderate disease (stage I–III) with age ranging from 30–70 years were recruited. COPD smokers were classified based on smoking for at least 10 pack years and those patients who left smoking for at least 1 year were categorized as COPD reformed smokers. All patients with a history of respiratory disorders other than COPD, active inflammatory disorders, previous lung volume reduction surgery/lung transplantation, intake of steroids over past 1 month, diabetes, exacerbations and infections for past 1 month were excluded. All patients were on medication. Age-matched healthy male controls were recruited as non-smokers (smoking history of <10 cigarettes in lifetime) and smokers (smoking at least 10 pack years) having normal spirometric values (FEV1/FVC > 0.70, FEV1 > 80% predicted, FVC > 80% predicted). Any history of respiratory disorders/active inflammatory disorders, intake of steroids over past 1 month and diabetes were excluded.

### 2.2. Sample Size Estimation

For the present study, sample size was computed to compare mean Treg cells between controls and each of the 2 groups of COPD patients, based on the following assumptions: (i) Mean ± SD of Treg in control and COPD group—5.33 ± 0.83* and 11.94 ± 0.38 [8], (ii) Confidence level—95%, (iii) Power of this study—90%. Even after considering multiple comparisons (control vs. COPD separately), 10 subjects in each group were considered to be adequate enough to detect above anticipated difference.

### 2.3. Anthropometric Measurements

Demographic features including age, sex, height and weight were noted. BMI was calculated, followed by COPD assessment test (CAT) scoring. Disease characteristics such as time since onset of symptoms, severity and medications taken were noted.

### 2.4. Spirometry

The procedure was conducted according to the guidelines given by ATS/ERS. Lung volumes and capacities were estimated using a dry rolling spirometer (Spiroair, Medisoft, PK Morgan Ltd., Kent, UK). The spirometer was calibrated using a three-liter syringe to ensure that it recorded accurate values before its use. FEV_1_ and FVC have been reported as the highest values from acceptable and reproducible trial.

### 2.5. Collection of Human Peripheral Blood Samples

After the recruitment, 8 mL peripheral blood was drawn from COPD patients and controls. For peripheral blood mononuclear cells (PBMCs) isolation, blood was immediately transferred to EDTA vial (Cat# 455036, Greiner Bio-one GmbH, Kremsmunster, Austria) to prevent clotting. Furthermore, for obtaining serum, blood was also transferred to plain vial (Cat# 3165894, BD Vacutainer, Franklin Lakes, NJ, USA).

### 2.6. Sample Processing

#### 2.6.1. Immunostaining

Peripheral blood mononuclear cells (PBMCs) were isolated via density gradient centrifugation using Histopaque. After the isolation, cells were counted using Neubauer hemocytometer. Optimization was performed by antibody titration and appropriate controls including isotype staining, and fluorescence minus one (FMO) controls were used. For antibody staining, PBMCs (1 × 10^6^) were divided and washed with 1 mL PBS at 300× *g* for 10 min. Cells were resuspended in 100µL cold cell staining buffer (#Cat 420201, Biolegend, San Diego, CA, USA). Titrated amount of antibodies for T cells (CD3-APC fire 750 (#Cat 300469, Biolegend), CD4-FITC (#Cat 317407, Biolegend), CD8-PerCp/Cy5.5 (#Cat 301031, Biolegend)), dendritic cells (CD11c-APC (#Cat 337207, Biolegend), CD123-PE (#Cat 306005, Biolegend), HLADR PerCP (#Cat 307627, Biolegend), Lin 1-FITC (#Cat 348801, Biolegend)), monocytes (CD14-APC (#Cat 325607, Biolegend)), and T regulatory cells (CD4-FITC (#Cat 317407, Biolegend), CD25-APC (#Cat 302609, Biolegend), CD45RA-PeCy7 (#Cat 304125, Biolegend), CD127-BV421 (#Cat 351309, Biolegend) were added in their respective tubes. Cells in Treg tube were fixed in 1 mL of 4 percent paraformaldehyde (#Cat 420801, Biolegend) and washed with 1 mL cold cell staining buffer. Following fixation, cells were permeabilized with 1 mL FoxP3 perm buffer (#Cat 421402, Biolegend) and washed. After that, intracellular FoxP3-PE (#Cat 320107, Biolegend) antibody was added and incubated at 4 °C for 45 min. Cells were washed twice with cold cell staining buffer before being resuspended in 400 µL PBS. Data were acquired on flow cytometer.

#### 2.6.2. Secretory Profiling

Secretory profiling was conducted using LEGENDplex human essential immune response panel (13-plex: 740930). It is a fluorescence-encoded bead-based multiplex assay panel that allows for simultaneous quantification of 13 cytokines essential for immune response such as IL-4, IL-2, CXCL10 (IP-10), IL-1β, TNF-α, CCL2 (MCP-1), IL-17A, IL-6, IL-10, IFN-γ, IL-12p70, CXCL8 (IL-8), and free active TGF-β1.

### 2.7. Statistical Analysis

Data were analyzed using GraphPad Prism (version 9.0). Subgroup differences were assessed using Kruskal–Wallis H test; in case of significant results, post hoc Dunn’s tests were conducted to further explore differences among groups. Data have been represented as median (interquartile range), and *p*-value less than 0.05 was considered statistically significant. Correlation analysis was performed depending on the distribution of the data.

## 3. Results

Overall, 41 participants were enrolled in this study. Immunophenotyping was performed to examine the baseline profile of immune cells, for which 18 COPD patients and 23 controls were recruited and demographic details of the same have been depicted in Table 1. On spirometric analysis, FEV_1_ was found to be significantly reduced in COPD smokers as compared to COPD reformed smokers (*p* < 0.0001).

FMO controls were used to discriminate between positive and negative population. Then, data were analyzed using FLOWJO V10.1 (Tree Star, Ashland, OR, USA). During analysis, ungated events were initially plotted in FSC-A × SSC-A plots to identify morphologically similar cells, followed by FSC-A × FSC-H plot for selection of single cells and exclusion of cell aggregates located far from the main diagonal. These cells were then gated for cell surface and intracellular markers depending on the cell type.

Kruskal–Wallis testing with post-hoc Dunn’s multiple comparison test were used for statistical analysis, and *p*-value < 0.05 was considered to be statistically significant. One asterisk (*) indicates a *p*-value < 0.05; two asterisks (**) indicate a *p*-value < 0.01; three asterisks (***) indicate a *p*-value < 0.001 and four asterisks (****) indicate a *p*-value < 0.0001.

### 3.1. T Cells

**Figure 1:**
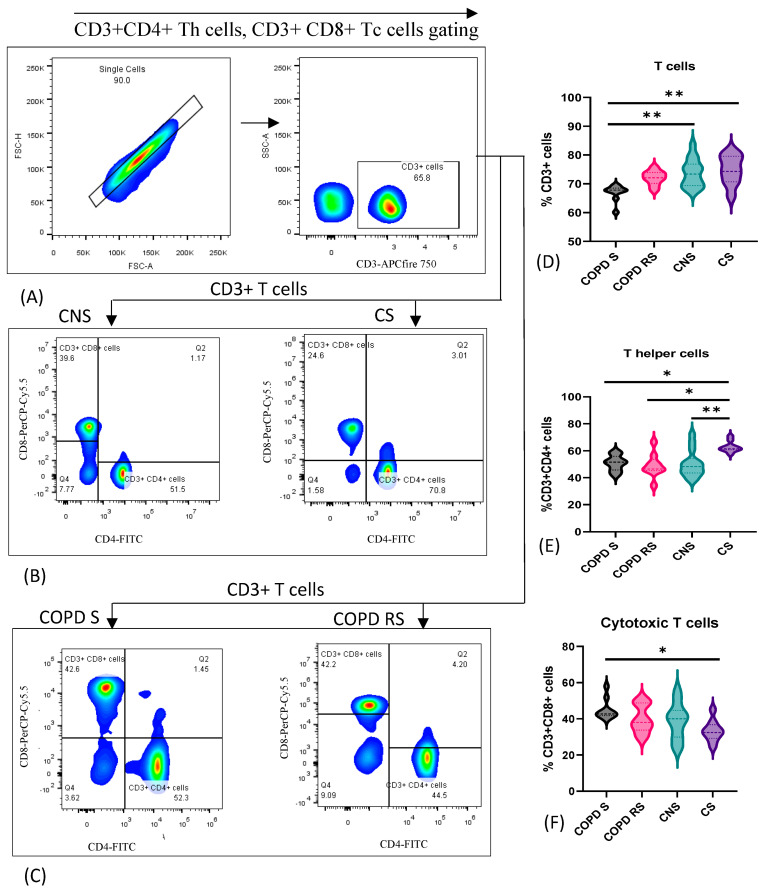
Representative pseudocolor plots showing sequential gating strategy used to identify T cell subtypes (**A**) Gating of CD3+ T cells from single cells. (**B**) T helper cells (CD4+) and cytotoxic T cells (CD8+) were gated from CD3+ T cells in control non-smoker (CNS) and control smoker (CS), (**C**) COPD smoker (COPD S) and COPD reformed smoker (COPD RS). Violin box plots showing (**D**) significantly lower frequency of CD3+ T cells in COPD S as compared to CNS and CS. (**E**) significantly lower frequency of CD4+ T cells in COPD S, COPD RS and CNS as compared to CS (**F**) significantly higher frequency of CD8+ T cells in COPD S as compared to CS. One asterisk (*) *p*-value < 0.05; two asterisks (**) *p*-value < 0.01.

T cell subsets were identified by the expression of CD3, CD4 on T helper cells and CD3, CD8 on cytotoxic T cells. Data have been represented as pseudocolor plots showing sequential gating strategy used to identify T cell subtypes (Figure 1). (A) CD3+ T cells were gated from single cells. (B) T helper cells (CD4+) and cytotoxic T cells (CD8+) were further gated from CD3+ T cells in control non-smoker (CNS) and control smoker (CS), (C) COPD smoker (COPD S) and COPD reformed smoker (COPD RS). On comparison between the subgroups, (D) significantly lower frequency of CD3+ T cells were observed in COPD S as compared to CNS (*p* < 0.01) and CS (*p* < 0.01), and (E) CD4+ T cells were significantly lower in COPD S (*p* < 0.05), COPD RS (*p* < 0.05) and CNS (*p* < 0.01) as compared to CS. However, (F) CD8+ T cells were elevated in COPD S as compared to CS (*p* < 0.05).

### 3.2. Classical Dendritic Cells

**Figure 2:**
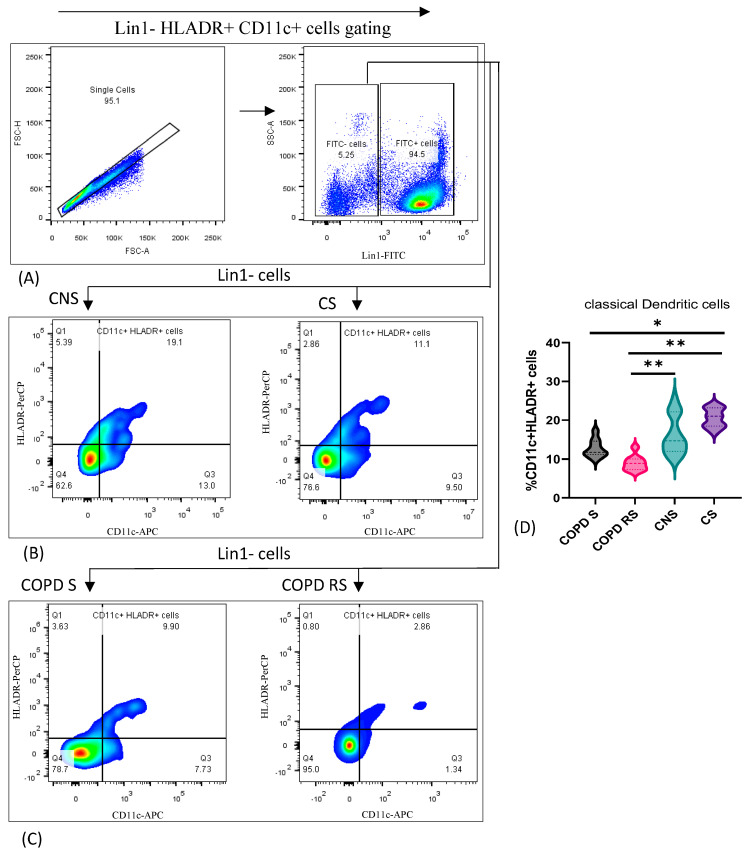
Representative pseudocolor plots showing sequential gating strategy used to identify classical Dendritic cells (cDCs) (**A**) shows gating of Lin1-cells from single cells. (**B**) CD11c+ HLADR+ cells in control non-smoker (CNS) and control smoker (CS), (**C**) COPD smoker (COPD S) and COPD reformed smoker (COPD RS). Violin box plots showing (**D**) significantly lower frequency of cDCs in COPD S as compared to CS and COPD RS as compared to CNS and CS. One asterisk (*) *p*-value < 0.05; two asterisks (**) *p*-value < 0.01.

Classical dendritic cells were identified by the expression of Lin1, CD11c and HLADR. Data have been represented as pseudocolor plots showing sequential gating strategy used to identify cDCs (Figure 2). After gating for lymphocytes and monocytes together, (A) Lin1-cells were gated from single cells. (B) CD11c+ HLADR+ cells were further gated from single cells in control non-smoker (CNS) and control smoker (CS), (C) COPD smoker (COPD S) and COPD reformed smoker (COPD RS). On subgroup analysis, (D) significantly lower frequency of cDCs were found in COPD S as compared to CS (*p* < 0.05) and COPD RS as compared to CNS (*p* < 0.01) and CS (*p* < 0.01).

### 3.3. Plamacytoiddendritic Cells

**Figure 3:**
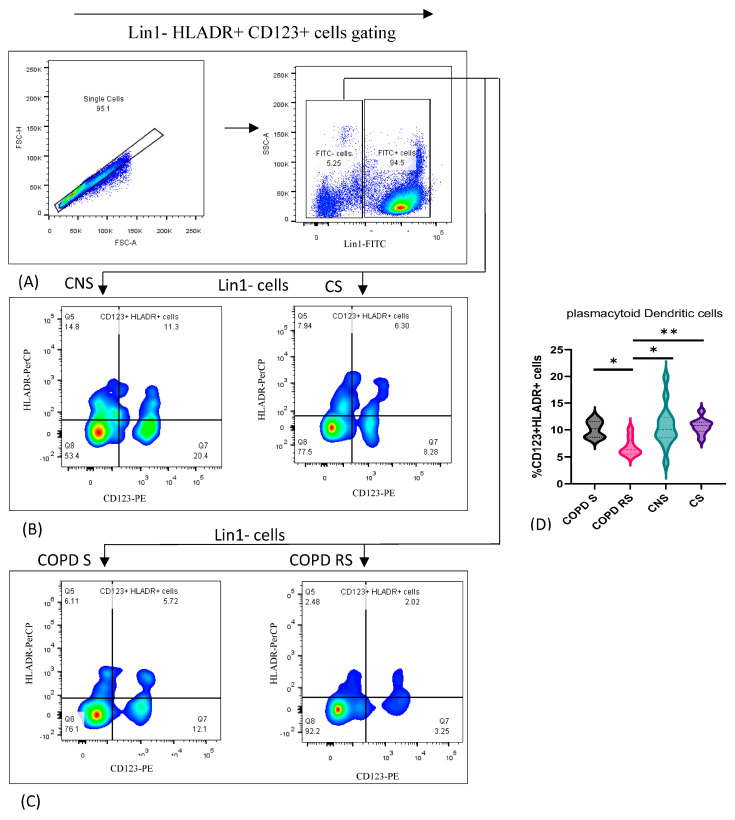
Representative pseudocolor plots showing sequential gating strategy used to identify plasmacytoid Dendritic cells (pDCs) (**A**) shows gating of Lin1-cells from single cells. (**B**) CD123+ HLADR+ cells in control non-smoker (CNS) and control smoker (CS), (**C**) COPD smoker (COPD S) and COPD reformed smoker (COPD RS). Violin box plots showing (**D**) significantly lower frequency of pDCs in COPD RS as compared to COPD S, CNS and CS. One asterisk (*) *p*-value < 0.05; two asterisks (**) *p*-value < 0.01.

Plasmacytoid dendritic cells were identified by the expression of Lin1, CD123 and HLADR. Data have been represented as pseudocolor plots showing sequential gating strategy used to identify pDCs (Figure 3). After gating for lymphocytes and monocytes together, (A) Lin1-cells were gated from single cells. (B) CD123+ HLADR+ cells were further gated from single cells in control non-smoker (CNS) and control smoker (CS), (C) COPD smoker (COPD S) and COPD reformed smoker (COPD RS). On subgroup analysis, (D) significantly lower frequency of pDCs were found in COPD RS as compared to COPD S (*p* < 0.05), CNS (*p* < 0.05) and CS (*p* < 0.01).

### 3.4. T Regulatory Cells

**Figure 4:**
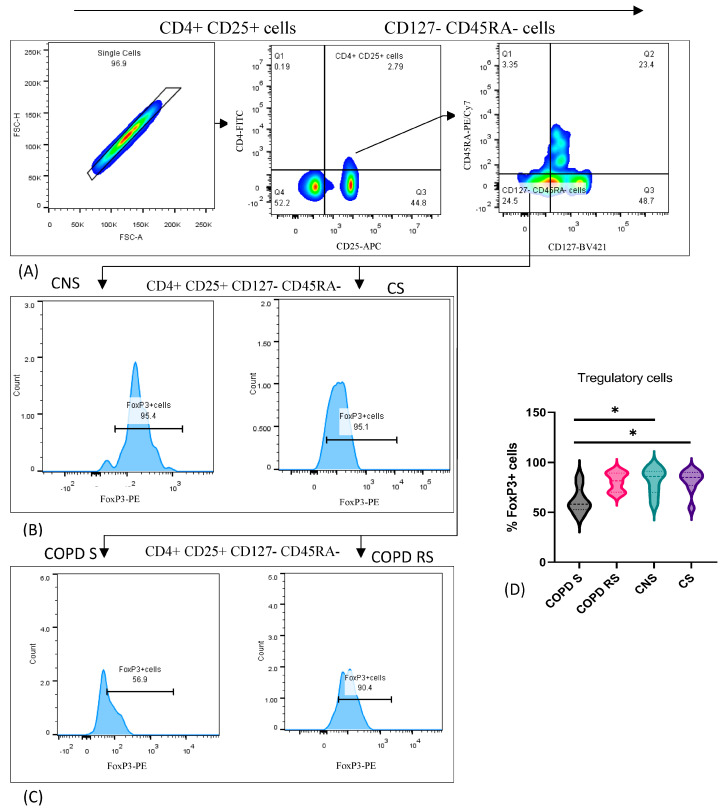
Representative pseudocolor plots and histograms showing sequential gating strategy used to identify T regulatory cells (**A**) shows gating of CD4+CD25+ T cells from single cells and CD127-CD45RA- cells from CD4+CD25+ cells (**B**) FoxP3+ cells in control non-smoker (CNS) and control smoker (CS), (**C**) COPD smoker (COPD S) and COPD reformed smoker (COPD RS). Violin box plots showing (**D**) significantly lower frequency of Tregs in COPD S as compared to CNS and CS. One asterisk (*) *p*-value < 0.05.

T regulatory cells were identified by the expression of CD4, CD25, CD45RA, CD127, and FoxP3. Data have been represented as pseudocolor plots and histograms showing sequential gating strategy used to identify Tregs (Figure 4). After gating for lymphocytes, single cells were gated: (A) shows gating of CD4 + CD25+ T cells from single cells and CD127-CD45RA- cells from CD4 + CD25+ cells; (B) furthermore, FoxP3+ cells were gated in control non-smoker (CNS) and control smoker (CS), (C) COPD smoker (COPD S) and COPD reformed smoker (COPD RS). Subgroup analysis showed significantly lower frequency of Tregs in COPD S as compared to CNS (*p* < 0.05) and CS (*p* < 0.05).

### 3.5. Monocytes

**Figure 5:**
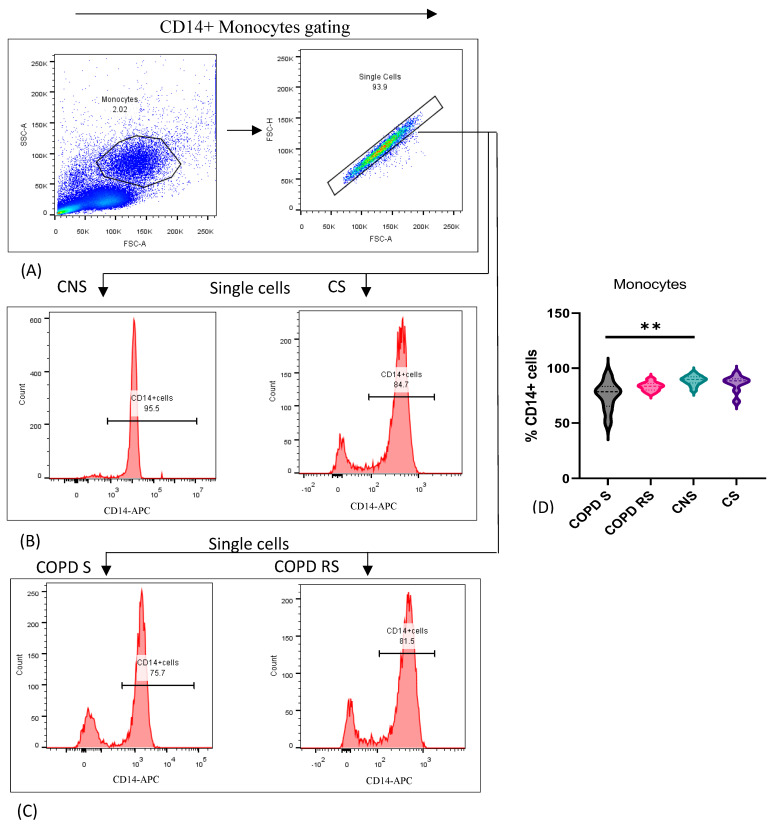
Representative pseudocolor plots and histograms showing sequential gating strategy used to identify Monocytes (**A**) shows gating of single cells from monocytes in FSC × SSC plot. (**B**) CD14+ cells in control non-smoker (CNS) and control smoker (CS), (**C**) COPD smoker (COPD S) and COPD reformed smoker (COPD RS). Violin box plots showing (**D**) significantly lower frequency of monocytes in COPD S as compared to CNS. Two asterisks (**) *p*-value < 0.01.

Monocytes were identified by the expression of CD14 and data have been represented as pseudocolor plots and histograms showing sequential gating strategy used to identify monocytes (Figure 5). After gating for monocytes, single cells were gated: (A) shows gating of single cells from monocytes in FSC x SSC plot; (B) CD14+ cells were further gated in control non-smoker (CNS) and control smoker (CS), (C) COPD smoker (COPD S) and COPD reformed smoker (COPD RS). On comparison between subgroups, COPD S had significantly lower frequency of monocytes as compared to CNS (*p* < 0.01).

### 3.6. Serum Cytokine Levels in COPD Patients and Controls

**Figure 6:**
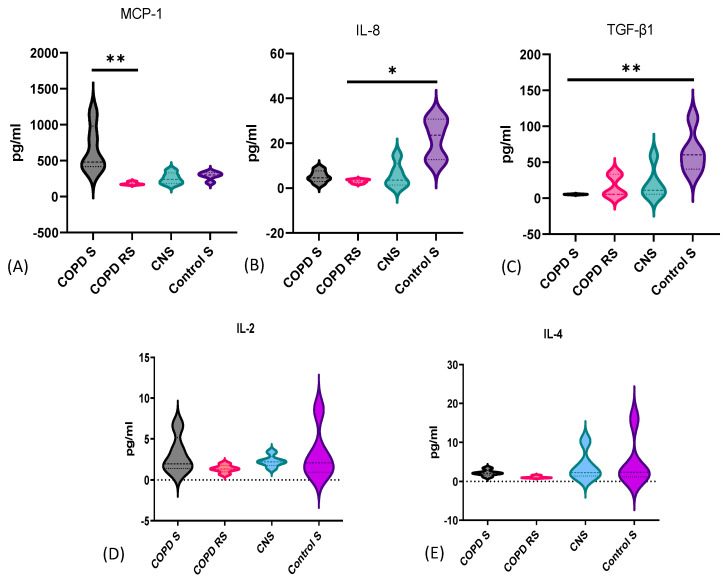
Violin box plots showing (**A**) elevated levels of MCP-1 in COPD S as compared to COPD RS, (**B**) lower levels of IL-8 in COPD RS as compared to CS, (**C**) lower levels of TGFß1 in COPD S as compared to CS, (**D**,**E**) IL-2 and IL-4 were comparable between subgroups. COPD S–COPD smoker, COPD RS—COPD reformed smoker, CNS—control non-smoker, CS—control smoker. One asterisk (*) *p*-value< 0.05; two asterisks (**) *p*-value< 0.01.

Serum samples of 10 COPD patients and 10 controls were analyzed for the quantification of cytokines. Data have been represented as violin box plots showing elevated levels of MCP-1 in COPD S as compared to COPD RS (*p* < 0.01); lower levels of IL-8 were observed in COPD RS as compared to CS (*p* < 0.05) and reduced TGFß1 in COPD S as compared to CS (*p* < 0.01) (Figure 6). Though 13 cytokines were analyzed, only 3 cytokines were significantly different and other cytokine levels were comparable between the subgroups (Table 2).

### 3.7. Correlation Analysis between Peripheral Immune Cells and Serum Cytokines

**Figure 7:**
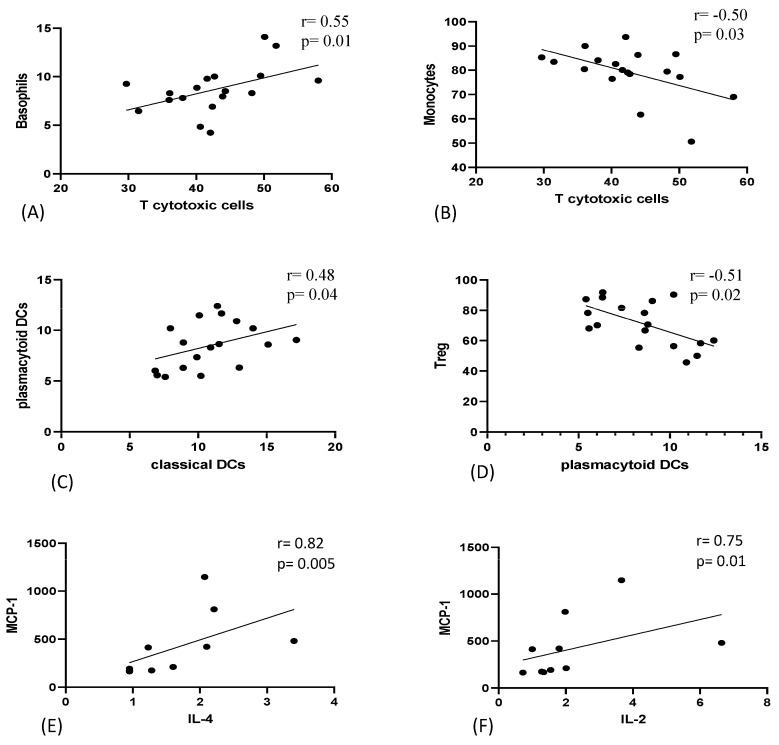
Correlation of immune cells and serum cytokines in peripheral circulation of COPD patients. (**A**) Positive correlation of T cytotoxic cells with Basophils. (**B**) Negative correlation of T cytotoxic cells with monocytes. (**C**) Positive correlation of classical dendritic cells with plasmacytoid dendritic cells. (**D**) Negative correlation of plasmacytoid dendritic cells with Tregs. (**E**) Positive correlation of IL-4 with MCP-1. (**F**) Positive correlation of IL-2 with MCP-1.

Spearman correlation between peripheral immune cells and serum cytokines was performed on 18 COPD patients (smokers and reformed smokers). T cytotoxic cells were positively correlated with basophils (r = 0.55) and negatively correlated with monocytes (r = −0.50). pDCs were positively correlated with cDCs (r = 0.48) and negatively correlated with Tregs (r = −0.51). Furthermore, IL-4 (r = 0.82) and IL-2 (r = 0.75) were positively correlated with MCP-1 (Figure 7). No significant correlation was found with any of the spirometric parameters.

## 4. Discussion

COPD is a complex and multi-factorial disease of the lung with pulmonary and extrapulmonary manifestations [9]. A number of mechanisms have been involved in the development of COPD; this inflammatory state is a self-perpetuating process that can last for years after smoking cessation [7]. Therefore, COPD has been characterized by a shift from the non-specific innate immune response that every smoker has, to an adaptive immune response with an autoimmune component [7]. In this study, one aspect of the complex inflammatory pathway that is known to be involved in the regulation of the immune response in COPD has been investigated.

To evaluate and quantify the effects of COPD symptoms on the health status of these patients, a COPD assessment test (CAT) was administered. In the present study, a higher CAT score was observed in COPD smokers as compared to reformed smokers. Similarly, a higher CAT score has been reported in COPD smokers with lower health-related quality of life and was also associated with severity of airflow limitation [10].

### 4.1. Immunophenotyping

#### 4.1.1. T cell Subsets in COPD

(a) CD4 helper T cells in COPD

In the present study, a significant decrease in the frequency of CD4+ T cells was found in COPD smokers, reformed smokers and control non-smokers as compared to control smokers. Similarly, lower blood CD4+ T cells along with decline in the CD4+ central memory, CD4+ effector memory, and CD4 + CD146+ T-cell subsets have been reported in COPD patients (current smokers and reformed smokers) as compared to controls [11,12]. In contrast, higher proportions of CD4 + IFNγ+ T cells in the circulation and peripheral airways of smokers with COPD as compared to controls have been observed [13]. In addition, no significant differences in CD4+ T cell subsets including cytokine profiles of CD4+ T cells have been reported in patients with COPD [14]. Despite the differences observed, the convergence of data denotes a potential dysregulation in blood CD4+ T cells in our cohort of patients and the decrease in the frequency could be attributed to the inability of these cells to mount appropriate immune response and/or having an exhaustive phenotype in COPD with disease progression.

(b) CD8 cytotoxic T cells in COPD

In the present study, a higher frequency of CD8+ T cells was found in COPD smokers as compared to control smokers; similar results were observed by Hodge et al., 2007 [15]. Furthermore, higher proportions of CD8+ T cells were found in bronchial biopsies [15], peripheral airways, lung parenchyma, and even paratracheal lymph nodes [16]. However, lower percentages of CD8+ T cells were observed in smokers with and without COPD as compared to controls [17]. In addition, no difference was reported in CD8+ T cell count in COPD patients and controls [18,19]. Also, cigarette smoking has been associated with increased percentages of CD8+ T cells in peripheral circulation of smokers and COPD patients [20] as compared to non-smokers [21]. Since in the present study an increase in the number of CD8+ T cells was found specifically in COPD smokers compared to control smokers, the observed increase might be due to COPD pathology alone.

The increase in CD8+ cytotoxic T cells might contribute to COPD pathogenesis through their cytotoxic effect along with the release of their associated pro-inflammatory cytokines. Since COPD has been known to have an autoimmune component with auto- reactive CD8+ T cells [22], one might argue that the increased CD8+ T cells along with lower Tregs observed in the present study might be facilitating the autoimmunity leading to tissue destruction. Also, in the present study, CD8+ T cells were found to have positive correlation with basophils implying a possible role of these cells in increased frequency of cytotoxic T cells.

(c) Regulatory T cells in COPD

Tregs are negative regulators of the immune system that help in maintaining homeostasis and self-tolerance. Data on regulatory T cells in COPD patients are scarce and even contradictory. In the present study, the frequency of CD4 + CD25 + CD45RA-CD127-FoxP3+ Tregs was found to be significantly lower in COPD smokers as compared to control smokers and non-smokers. Similar results were observed, raising the possibility of these cells to play a crucial role in the pathogenesis of the disease. Also, lower levels of activated Tregs, resting Tregs, and significantly higher levels of pro-inflammatory cells have been reported in COPD [23,24]. However, smokers without COPD have been reported to have higher levels of Tregs, suggesting a potential Treg protective role against developing COPD [25].

Our findings, however, are in contrast with those of Barcelo et al., who found no differences in CD4 + CD25+ Tregs from peripheral blood between COPD and controls as well as Vargas-Rojas et al., who found higher levels of Treg cells in COPD and smoker subjects than in healthy controls [26,27]. Higher proportions of Tregs were also found in the pulmonary lymphoid follicles of severe COPD patients as compared to smokers and non-smokers [28]. Progression of COPD, despite an increase in the number, has been attributed to a potential impairment in the functional capacity of Tregs.

Furthermore, according to previous studies, the number of peripheral Tregs have been seen to increase with the patient’s age [29]; since the data in the present study indicate the contrary, one may speculate that COPD pathology might be primarily responsible for the observed results.

The decrease in the frequency of Tregs observed in the present study could be attributed to probable exhaustion of the anti-inflammatory responses despite disease progression and continued noxious exposure specifically in COPD smokers.

Based on these findings, we hypothesize that the persistent airway inflammation dominated by CD8+ T cells is caused by a reduction in the immunosuppressive Treg populations and an increase in pro-inflammatory cytokine, i.e., increase in serum MCP-1, induced by long-term exposure to noxious particles as well as decrease in CD4 + T cells. This leads to an intriguing conjecture about the interaction between lymphocyte subpopulations in the development of COPD. One may argue that the increase in the frequency of CD8+ T cells might not involve a concomitant increase in number of T-regulatory cells in COPD patients. The inability of T-regulatory cells to increase in number may be a mechanism for unregulated lung inflammation, facilitating CD8+ T cell-mediated positive feedback to enhance further CD8+ T cell influx. Also, in the present study, Tregs were found to have a negative correlation with inflammatory plasmacytoid dendritic cells.

#### 4.1.2. Dendritic Cells in COPD

In the present study, we found a significant decrease in classical dendritic cells in COPD smokers and reformed smokers as compared to control smokers and non-smokers. Plasmacytoid dendritic cells were also significantly lower in COPD reformed smokers as compared to COPD smokers, control smokers and non-smokers. Similarly, lower proportions of mature DCs (mDCs), a subtype of DCs [30] and circulating pDCs [31] have been observed in COPD compared to controls. Additionally, mature pDCs have been reported to produced more TNFα and IL-8 and become accumulated in lymphoid follicles of COPD patients in a GOLD stage-dependent manner [32]. Furthermore, COPD smokers were found to have less overall bronchial DCs as compared to non-smokers [33]. Also, it has been demonstrated that myeloid DC differentiation is impaired in small airways of COPD patients and current smokers as compared to never-smokers [34]. However, Kalathil et al. and Stoll et al. did not observe any differences in blood pDCs concentrations [35]. In addition, higher DC concentrations have been reported in the lung parenchyma [36] of COPD patients, indicating conflicting data on the DC levels in COPD. The decrease in the frequency of cDCs and pDCs observed in the present study could be attributed to plausible impairment in antigen presentation, leading to a probable decrease in activation of CD4+ T cells as evident by lower levels of CD4+ T cells and increased susceptibility to respiratory infections. Also, in the present study, cDCs and pDCs were found to be positively correlated with each other.

#### 4.1.3. Monocytes in COPD

In the present study, blood monocytes obtained from COPD patients were profiled with regard to the expression of CD14 and a significant decrease in CD14+ monocytes were observed in COPD smokers as compared to control non-smokers.

In contrast to our results, Silva et al. found elevated frequencies of intermediate monocytes (CD14+/CD16+) in COPD patients as compared to healthy donors. However, Cornwell et al. reported no change in the overall number of monocytes in patients with moderate COPD or controls, but there was a significant increase in the number of monocytes in patients with severe COPD [37]. Both phagocytosis and efferocytosis by macrophages have been shown to be perturbed by cigarette smoke as well as COPD exacerbation [38]. Despite the disparity in the frequency of circulating CD14 monocytes, it has been established from previous studies that monocyte migratory ability, cytokine production and phagocytosis might be hampered in COPD [39].

Therefore, the decrease in the frequency of CD14+ monocytes observed in the present study, despite increase in the serum concentrations of chemotactic factor MCP-1 (which is known to induce and recruit monocytes [40]), points towards potential functional impairment of these cells. In addition, one might speculate that the decreased CD4+ T count in COPD could be due to the inability of monocytes to activate and present antigen to CD4+ T cells. Also, the high proportions of CD8+ T cells found in the present study with probable dysfunctional monocytes could lead to impaired clearance of cellular debris. In addition, monocytes were found to have negative correlation with CD8+ T cells implying a possible role of these cells in reduced levels of monocytes. Furthermore, macrophages have been reported to accumulate in the airways of COPD patients and peripheral monocytes are presumed to replenish lung macrophages, which could also be one of the reasons for decreased monocytes in peripheral circulation.

The disparity seen in the frequency of peripheral immune cells in the present study from the previous literature could be a result of patient selection, i.e., only stable COPD patients were recruited, medications being used, severity of the disease, existence of various comorbidities and/or technical approach to identify various immune cells, i.e., cell surface and intracellular markers.

### 4.2. Serum Cytokine Levels in COPD Patients and Controls

(a) Monocyte chemoattractant protein-1 in COPD

In the present study, significantly elevated levels of serum MCP-1 were observed in COPD smokers as compared to COPD reformed smokers. In line with our results, Liu et al. found greater plasma MCP-1 concentrations in COPD patients than healthy controls [41]. In a subgroup of COPD patients with prevalent emphysema, the blood levels of MCP-1 were significantly elevated, pointing to a potential function of this molecule in the lung changes associated with this phenotype of COPD [42]. In addition, the severity of COPD has been positively correlated with an increase in MCP-1, which is thought to be a factor in the systemic comorbidities associated with COPD [42].

The elevated levels of MCP-1 observed in the present study could be speculated to be caused by mediators that “overspill” from the lungs, since the inflammation that manifests is not limited to the lungs [42]. Also, the increase was significant in COPD smokers with chronic noxious exposure and after smoking cessation, MCP-1 levels were found to be lower in COPD reformed smokers. In addition, MCP-1 was found to be positively correlated with IL-2 and IL-4, indicating a plausible role of these cytokines in elevated levels of serum concentrations of MCP-1 in the current study.

(b) Interleukin-8 in COPD

In the present study, we found a significant decrease in the levels of serum IL-8 of COPD reformed smokers as compared to control smokers. Earlier studies have reported higher levels of IL-8 in induced sputum with increase in the frequency of exacerbation in COPD patients [43]. Additionally, Xie et al. [44] reported that as compared to patients with stable COPD and healthy controls, serum IL-8 was significantly greater during COPD exacerbations. Also, serum IL-8 were correlated with the severity of COPD [45] and higher levels of IL-8 were found in advanced stages of the disease [46].

The disparity in data might be due to the recruitment of stable COPD patients in the present study as compared to patients with acute exacerbation or advanced disease in other studies. Being a proinflammatory marker, the decrease in reformed smokers might be attributed to the remission of active noxious smoke exposure in these patients.

(c) Transforming growth factor beta-1 in COPD

In the present study, a significant decrease in serum TGFß1 was found in COPD smokers as compared to control smokers. Similarly, Kamio et al. found significantly reduced serum TGFß1 levels as emphysema progressed in COPD patients and attributed the disappearance of small airways to the accelerated formation of emphysema [47]. However, Mak et al. reported elevated plasma levels of TGFß1using ELISA in COPD as compared to healthy controls, suggesting that this might be a consequence of the inflammatory process associated with COPD, and TGFß1 might be secreted by the inflammatory cells in response to tissue injury [48]. There have been conflicting reports on the levels of TGFß1 in the blood of COPD patients and the disparity could be due to patient selection criteria and technical approach to quantify cytokine. In the present study, the decrease could be attributed to lower Treg frequency and reduced cytokine-mediated suppression by Tregs, pointing in a direction towards systemic immune dysfunction.

Therefore, the increase in the serum concentrations of MCP-1 and decrease in TGFß1 specifically in COPD smokers implies a potential contribution of noxious exposure to systemic inflammation observed in COPD. The decrease in IL-8 levels further indicates a plausible remission of inflammation in COPD reformed smokers on smoking cessation.

## 5. Conclusions

**Figure 8:**
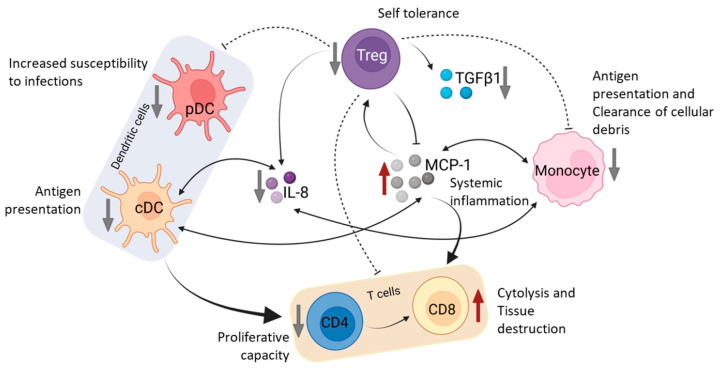
Schematic diagram depicting putative mechanism involving plausible interaction, leading to imbalance in the subsets of peripheral immune cells along with their soluble mediators in COPD. Though cigarette smoke is considered to be the primary risk factor, the progression of the disease has been attributed to dysfunction in regulatory mechanisms that include weak anti-protease and anti-oxidant activities, and maladaptive immune modulation [49,50]. It is still unclear whether systemic inflammatory markers represent a “spillover” from inflammation in the lungs, or if they are a parallel aberration or linked to a concomitant disease that affects the lungs. The decrease in dendritic cells and monocytes observed in the present study might result in decreased antigen presentation leading to decreased helper T cells. The lower numbers of helper T cells along with plasmacytoid dendritic cells might predispose COPD patients to bacterial/viral infections. However, on the other hand, the decrease in anti-inflammatory Tregs along with serum TGFß1, despite increase in proinflammatory cytotoxic T cells and serum MCP-1 might cause the observed imbalance in the immune response. It is worth noting that there also exists a network of crosstalk between the immune cells and the cytokines leading to the observed pathology in COPD (Created with BioRender.com).

Any alterations in the levels of immune cells in peripheral blood could be hypothesized to have a significant impact on a number of changes described in COPD, including the following (Figure 8): (a) persistence of inflammation (due to elevated levels of MCP-1 and CD8+ T cells), (b) increased susceptibility to viral infections (due to decrease in CD4+ T cells and pDCs), (c) decreased antigen presentation (due to decrease in cDCs and monocytes), (d) impaired clearance of debris (due to decrease in monocytes), (e) decreased immune tolerance (due to decrease in Tregs and TGFß1).

## Figures and Tables

**Table 1:** Demographic data of the participants.

Parameters	COPD S (*n* = 9)	COPD RS (*n* = 9)	CNS (*n* = 14)	CS (*n* = 9)
Age (years)	59.5 ± 5.0	56.3 ± 6.0	55.71 ± 7.7	58.3 ± 9.7
Height (cm)	165.0 ± 3.8	162.4 ± 6.2	167.2 ± 3.5	164.2 ± 5.7
Weight (Kg)	60.4 ± 10.0	66.7 ± 10.7	73.6 ± 7.5	71.2 ± 11.6
BMI (Kg/m^2^)	22.1 ± 3.5	25.3 ± 4.4	26.3 ± 2.7	26.3 ± 3.9
CAT score (0–40)	23.7 ± 5.7	17.5 ± 4.1	NA	NA
Smoking status	Smoker	Reformed smoker	Non-smoker	Smoker
Smoking pack years	20.4 ± 5.6	-	-	21.5 ± 4.5
FEV_1_ %pred *	42.6 ± 4.0	60.4 ± 5.3	>80	>80
FVC %pred	63.2 ± 4.8	65.1 ± 5.7	>80	>80
FEV_1_/FVC	59.8 ± 3.0	67.3 ± 5.7	>70	>70

Data have been expressed as mean ± SD, COPD S—COPD smoker, COPD RS—COPD reformed smoker, CNS—control non-smoker, CS—control smoker, NA—not applicable, BMI—body mass index, CAT—COPD assessment test, and asterisk indicates significant difference between the groups.

**Table 2:** Cytokine levels in serum of COPD patients and controls.

S.No.	Cytokines	COPD S	COPD RS	CNS	CS	*p*-Value
1	IL-4	2.10 (2.80–1.65)	0.95 (1.44–0.95)	2.28 (7.23–1.40)	2.39 (9.54–1.17)	0.08
2	IL-2	1.98 (5.15–1.40)	1.34 (1.78–1.00)	2.23 (2.87–1.79)	2.10 (5.90–0.93)	0.26
3	IP-10	60.10 (388.70–32.74)	68.29 (126.50–25.25)	43.34 (68.42–38.95)	60.96 (77.72–38.59)	0.97
4	IL-ß1	1.22 (1.92–1.22)	2.28 (5.31–1.22)	1.73 (2.57–1.22)	1.22 (11.95–1.22)	0.58
5	TNFα	ND	ND	ND	ND	-
6	MCP-1	480.20 (980.40–416.70)	174.40 (201.40–166.0)	237.90 (330.10–182.90)	301.40 (333.80–242.3)	0.002 *
7	IL-17	ND	ND	ND	ND	-
8	IL-6	2.90 (3.15–1.56)	1.44 (4.90–0.71)	0.79 (3.16–0.71)	9.67 (33.09–1.52)	0.43
9	IL-10	ND	ND	ND	ND	-
10	IFN-γ	ND	ND	ND	ND	-
11	IL-12P70	0.60 (4.53–0.60)	0.60 (1.08–0.60)	0.60 (2.69–0.66)	3.21 (10.14–0.60)	0.33
12	IL-8	4.58 (7.68–2.98)	3.12 (3.83–2.30)	3.58 (10.97–1.36)	23.55 (30.78–12.67)	0.01 *
13	TGFß1	5.21 (5.21–5.21)	5.21 (33.16–5.21)	10.96 (39.95–5.21)	60.30 (89.29–40.23)	0.005 *

Data have been expressed as median (IQR), COPD S—COPD smoker, COPD RS—COPD reformed smoker, CNS—control non-smoker, CS—control smoker, ND—not detectable, IL—Interleukin, TNFα—Tumor necrosis factor alpha, MCP-1—Monocyte chemoattractant protein, IFN-γ—Interferon gamma, TGFß1—Transforming growth factor beta. One asterisk (*) *p*-value < 0.05.

## Data Availability

Data unavailable due to privacy issues.
